# Hurricane-induced disturbance increases genetic diversity and population admixture of the direct-brooding isopod, *Gnathia marleyi*

**DOI:** 10.1038/s41598-020-64779-7

**Published:** 2020-05-26

**Authors:** J. Andrés Pagán, Ana Veríssimo, Paul C. Sikkel, Raquel Xavier

**Affiliations:** 1 CIBIO- Universidade do Porto, Centro de Investigção em Biodiversidade e Recursos Genéticos, Campus Agrário de Vairão, Rua Padre Armando Quintas, 4485-661 Vairão, Portugal; 2 Department of Biological Sciences and Environmental Sciences Program, Arkansas State University, PO Box 599, AR 72467 State University, USA; 3 Water Research Group, Unit for Environmental Sciences and Management, North-West University, Potchefstroom, South Africa

**Keywords:** Zoology, Structural variation, Marine biology, Phenology

## Abstract

Severe disturbances can substantially alter eco-evolutionary processes and dynamics. While the impacts of catastrophic events on the biophysical attributes of communities are sometimes assessed, their effects on the genetic patterns of species remain poorly understood. To characterize how severe disturbances impact species at the molecular level, we examined the effects of the most energetic North Atlantic hurricane season in 50 years on the genetic diversity and structure of a dispersal-limited isopod, *Gnathia marleyi*. We sequenced a portion of the cytochrome oxidase I gene for 432 gnathiids, collected from six localities, ranging from western Puerto Rico to St John, US Virgin Islands. Importantly, multiple years of pre-hurricane sample collection allowed us to characterize temporal genetic patterns under undisturbed conditions and detect the changes subsequent to the 2017 hurricanes. Our results revealed no change to genetic diversity or structure for the years prior to the 2017 hurricanes, with genetic structure occurring at the local and regional levels, with three main clusters corresponding to Southwest Puerto Rico, East Puerto Rico, and the US Virgin Islands. However, directly following the 2017 hurricanes, genetic diversity increased at five of the six sampled localities. Additionally, we found a clear homogenizing effect prompted by increased shared genetic diversity among geographically distant regions and sites that resulted in substantially decreased among-region and among-site differentiation. Our work shows that severe disturbances caused by major tropical hurricanes facilitate gene-flow and increase overall genetic diversity and population admixture of dispersal limited coral reef species, potentially impacting the ecology and evolution of a key regional endemic.

## Introduction

A major focus in ecology has been to understand how environmental disturbances impact species and communities^1^. Disturbances are integral processes that can alter community assemblages, re-structure spatial distribution of species, and drive biodiversity patterns^2–5^. Severe disturbances often modify the physical landscape, potentially affecting habitat suitability and/or availability^6^. As a result, they can induce mortality and affect population connectivity, which are key biological processes that determine genetic diversity and structure of populations^7^. The effects of these perturbations can be observed at the molecular level by measuring changes in the spatial distribution of alleles and patterns of gene-flow; over long time scales these processes can ultimately affect population adaptation and viability^8,9^. For instance, wildfires and large volcanic eruptions can cause mortality and habitat fragmentation, leading to population bottlenecks and loss of genetic diversity^10,11^. However, if after a disturbance survival and gene-flow are high, and sites are re-colonized from multiple sources, the genetic diversity can be maintained or even increased^12,13^. Notably, much of disturbance ecology theory has been informed largely by terrestrial case studies while few empirical investigations have assessed the genetic signatures of severe disturbances in marine systems^14^.

Major tropical cyclones are amongst the most severe disturbances in marine ecosystems, particularly for shallow, coastal habitats, such as coral reefs^15,16^. Cyclones can damage the physical structure of coral reefs^17,18^, displace massive amounts of water^19^, diminish water quality^20^, and alter fish community assemblage^21–23^. The fact that major cyclones are difficult to anticipate, even at relatively short timescales, makes it difficult to conduct proper Before-After-Control-Impact (BACI) -type sampling needed to rigorously test their effects^24^. Nevertheless, a few studies have attempted to measure the effects of similar disturbances on the genetic structure of marine taxa. For example, Brante *et al*.^25^ found that a species’ capacity to disperse can greatly influence how it responds to major disturbance, however, they only measured changes to genetic structure following a major disturbance without sampling beforehand. Other studies that incorporated pre- and post- disturbance sampling have focused on species with high dispersal potential for which disturbance only impacted a small portion of a much wider distributional range (a mud snail and intertidal goby affected by a 2011 tsunami^26,27^). In another, study, pre and post-disturbance genetic structure was compared using different molecular markers with large time gaps before and after the purported disturbance (an estuarine fish impacted by a 2005 hurricane^28^). Thus, additional research is still required to understand the acute effects of severe environmental disturbances on the genetic diversity and structure of coastal marine species, especially for regional endemics with limited dispersal.

Gnathiid isopods (Peracarida) are a key functional group within the community of small, mobile, benthic marine invertebrates. Analogous to ticks and mosquitos, the larval stages feed on blood and body fluids of a wide range of benthic and demersal fishes^29^. While they occur from the intertidal to the abyss, they are particularly abundant in coral reef systems where they play a key role in cleaning symbiosis and other trophic interactions^30^. After feeding on a single host, first-stage juvenile gnathiids detach and shelter in the substrate where they molt into the next instar following a period of 5–7 days (in the tropics; temperate species require longer), upon which the feeding-molting cycle is repeated two additional times (Smit & Davies 2004, Tanaka 2007). After the third feeding, juveniles refuge in the benthos, where they metamorphose into adults. Unusual among parasites, adult gnathiids do not feed, and spend the entirety of their lives in the benthos. Males may live for a month or more after metamorphosis, however, females die after giving birth to about 30 live young^31^. Thus, tropical gnathiids can complete their life cycle in as little as 30 days^30,32,33^. While gnathiids can swim in short bursts, they cannot swim long distances and lack a pelagic dispersal phase (direct brooders). Although they attach to swimming hosts, their association is temporary (1–4 hours) and many of their known hosts have high site fidelity^34–38^, limiting the distance over which gnathiids can be dispersed through host-attachment^39^. The spatial genetic structure of gnathiid species has so far been considered only for geographically distant populations, at scales of more than 2000 km^40^; yet, the scale of genetic structure in dispersal-limited marine species is often much smaller^41^ and fine-scale population genetic structure of gnathiids remains unexplored.

Herein, we characterize the impacts of the 2017 hurricane season on the genetic diversity and structure of *Gnathia marleyi*^42^, a species of fish-parasitic gnathiid isopod endemic to the northeastern Caribbean, a region that is vulnerable to tropical cyclones. The 2017 North Atlantic hurricane season, which included two category five hurricanes, was one of the most energetically intensive of the past 50–100 years^43^ and caused a 25% decline of key benthic coral reef species^44^. We hypothesized that due to their limited innate dispersal ability and stable environmental conditions, pre-hurricane gnathiid populations would show a strong pattern of isolation by distance, with individuals collected at each locality being genetically differentiated. Following the hurricanes, we expected to find an increase in genetic diversity due to putative hurricane-induced migration among populations. Further, we expected increased gene flow to reduce population differentiation particularly among geographically close populations (e.g. at the intra-island level). Additionally, since the hurricane trajectories generally moved from east to west, we predicted the induced gene flow to be directional, with western populations receiving haplotypes from “upstream” eastern counterparts.

## Materials and Methods

**Figure 1:**
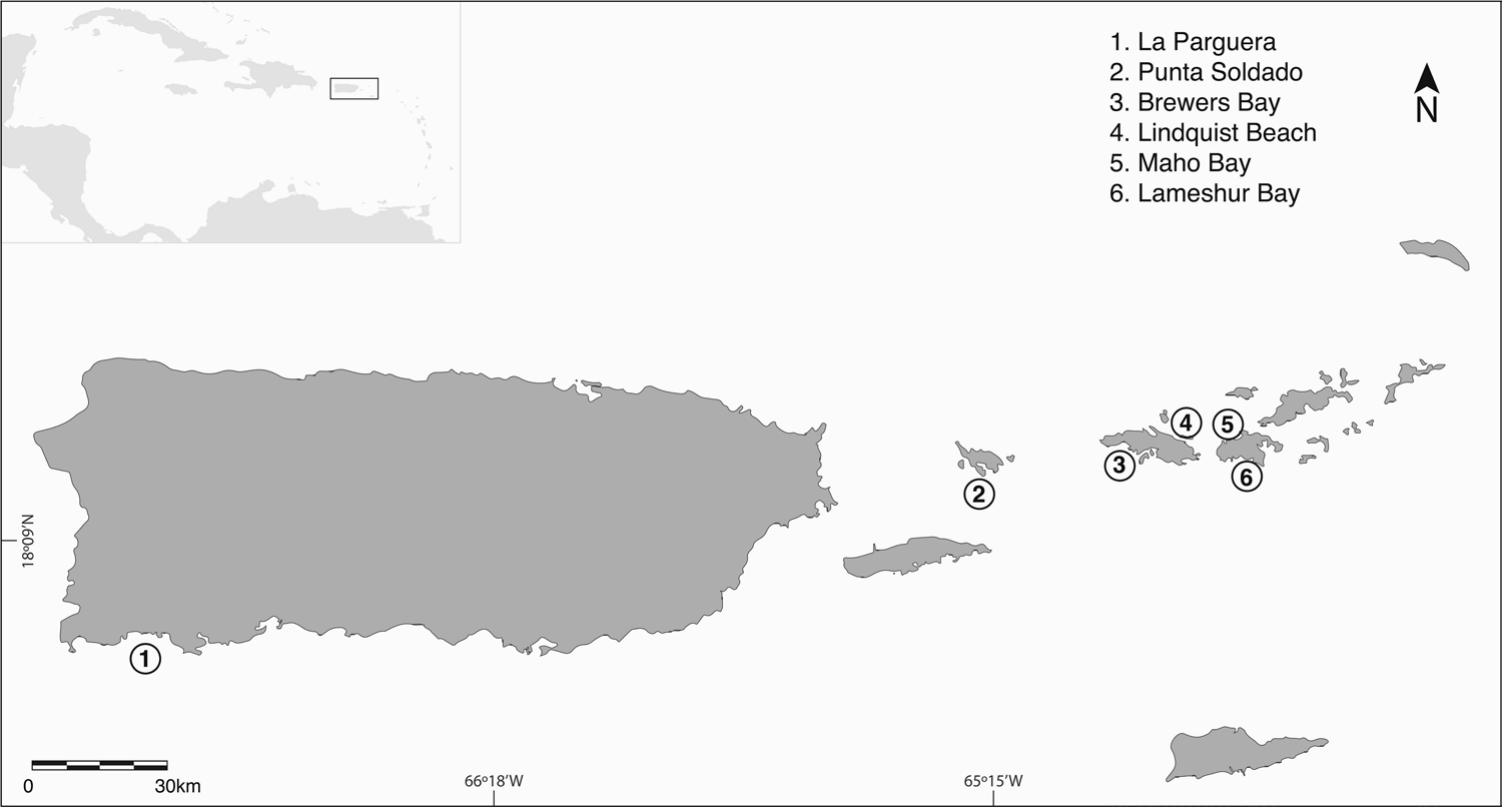
Map illustrating the spatial relationships between the sites sampled. Encircled numbers indicate the geographic location of each sampling site.

Gnathiids were collected between late spring and summer (May to August) of 2013–2018 from six sites in the Northeast Caribbean Sea, spanning from southwestern Puerto Rico (17°57′18.0″N 67°03′03.7″W) to southeastern St John, US Virgin Islands (18°18′58.6″N 64°43′18.8″W) (Fig. 1). La Parguera, Punta Soldado, and Lameshur Bay were each sampled multiple years prior to the hurricanes in order to measure variation in genetic diversity and structure under undisturbed conditions. Brewers Bay, Lindquist Beach, and Maho Bay were each sampled once before and once after the 2017 hurricane season. Individuals were collected from reef habitats between 3–10 m depth. Most samples were collected using light traps^45^ that were deployed before sunset and retrieved the following morning after sunrise. Upon retrieval, contents of light traps were filtered and visually scanned under 10x magnification to separate gnathiids from other organisms. When sampling with light traps was not possible, gnathiids were collected using live fish-baited cages placed on the reef^29^. Caged fish were set 30 minutes before sunset and retrieved 30 minutes before sunrise the following morning and gnathiids were then gently dislodged from the fish. All samples were preserved in 96% ethanol and stored at −20 °C.

In the laboratory, individual gnathiids were placed into 2.0 ml Eppendorf tubes, rinsed with distilled water, and incubated at 37 °C until completely dry. Once dry, gnathiids were pulverized inside the Eppendorf tubes using a steel rod and used for genomic DNA (gDNA) extraction with the Invitrogen PureLink genomic DNA mini extraction kit, following the manufacturer´s protocol (Invitrogen, Carlsbad, California). The gDNA was eluted into 55 µL working stocks with a final concentration of 5–10 ng/µL. Polymerase chain reactions (PCR) were performed to amplify a ~1200 base pair fragment of the cytochrome oxidase subunit I mitochondrial DNA (COI) using the primers crust-coix1f: ACTAATCACAARGAYATTGG and crust-coix1r: TAGTCTGAGTANCGTCGWGG^46^. PCR reactions were performed in 10 µL volumes consisting of 3.5 µl of water, 5.0 µL QIAGEN Taq PCR master mix (Qiagen, Valencia, CA), 0.5 µL of each primer (10 µM), and 0.5 µL gDNA. The PCR temperature profile included a step of initial denaturation at 95 °C for 15 minutes, followed by 35 cycles of 94 °C for one minute, 55 °C for one minute, 72 °C for one minute, and a final extension step at 72 °C for 10 minutes. Due to the low primer specificity, species-specific primers were newly designed (gmarCOI5for: GGGATTTTTAGAGAATGAGCA and gmarCOI5rev: TCCAAACCCTGGAAGGATA) using PrimerBLAST^47^ and targeting a 700 bp portion of COI. Final PCR reactions consisted of 3.5 µl of water, 5.0 µL QIAGEN master mix, 0.5 µL each of forward and reverse primer (10 µM), and 0.5 µL gDNA and the following temperature profile: initial denaturation at 95 °C for 15 minutes, 35 cycles of 94 °C for 45 s, 55 °C for 45 s, and 72 °C for 45 s, and final extension at 72° for 10 minutes. PCR products were sent for sequencing at GENEWIZ (Genewiz, Leipzig, Germany). Amplicon sequences were edited using CodonCode Aligner V.8.0.1 (Codon Code Corporation, Centerville, Massachusetts) and aligned using MAFFT^48^ with the default parameters. The multiple sequence alignment was then translated to amino acid sequences using MEGA^49^ to check for stop codons. A BLAST search was performed to validate that we had amplified correct DNA amplicon. *Gnathia marleyi* sequences diverged from Pacific congenerics by 31.5 to 36.2% similar to values observed among Pacific gnathiids^50^. The final alignment was truncated to 517 homologous base pairs.

Genetic diversity indices were estimated for each sample collection, including average number of pairwise differences (Pi), haplotype diversity, and nucleotide diversity, using Arlequin 3.5^51^. Haplotype evenness and the expected number of haplotypes (i.e. number of haplotypes standardized for sample size) were calculated using the poppr package in R^52^. Haplotype networks were constructed to visualize the spatial distribution of haplotypes in the samples collected before and after the hurricanes using TCS v1.21^53^ with a connection limit set to 95% and imported to tcsBU for graphical representation^54^. To assess the temporal variation of genetic diversity under undisturbed conditions, we performed permutational multivariate analysis of variance (PERMANOVA)^55^ considering only data collected before the storms. The effect of Year (year of sample collection) was tested for each molecular diversity index, with permutations constrained within Site (sample location). To test whether the 2017 hurricanes significantly altered genetic diversity, PERMANOVAs were performed for each molecular index with Disturbance (data from pre/post-hurricanes) and Site as factors (Disturbance x Site), and by constraining permutations within Site. For all analyses, Manhattan distances were used, and 10,000 permutations were performed. PERMANOVAs were performed in R using the function adonis implemented in the vegan package^56^.

Pairwise PhiST comparisons were used to assess temporal genetic differentiation within-site under undisturbed conditions, using only pre-hurricane samples for sites with multiple years of sampling (La Parguera, Punta Soldado, Lameshur Bay). To assess whether the 2017 hurricanes altered differentiation within-site and among-sites, pairwise PhiST tests were also performed using pre- and post-hurricane datasets. Since PhiST values within sites were not significantly different between years sampled prior to the 2017 hurricane season, all sample collections prior to 2017 hurricanes were pooled per site prior to estimation of pairwise PhiST values between pre- and post-hurricane datasets. Statistical significance of pairwise PhiST values were corrected for multiple tests with a Holm-Bonferroni correction^57^. Finally, an analysis of molecular variance (AMOVA) was used to test two population structure scenarios: a) complete panmixia, and b) population structure at the regional level, according the haplogroups recovered from the haplotype network and to the geographical proximity among sites (i.e. La Parguera-W. Puerto Rico; Punta Soldado-E. Puerto Rico; U.S. Virgin Islands). AMOVAs were performed separately for pre- and post- hurricane datasets to assess whether the amount of genetic variance explained by among-site and/or among-group differences changed after the 2017 hurricane season. PhiST estimates and AMOVAs were calculated in Arlequin 3.5 based on pairwise differences among haplotypes and 10,000 permutations of the data.

To test whether gnathiids are structured following a pattern of isolation by distance (IBD), a linear regression of the geographic distance between sampling locations onto pairwise PhiST values was performed. Geographic distances were square root transformed to normalize distributions, and analyses were run using R. The IBD test was performed for both pre-hurricane samples and post- hurricane samples.

Finally, to infer migration events and directionality of geneflow before and after the storms, we performed Monte Carlo resampling under a Bayesian framework using Geneclass2^58^. We selected the log_home/log_max assignment criteria to identify first-generation migrants and their likely population of origin with populations assigned according to sampling locality. Analyses were performed using the Bayesian method described in Rannala and Mountain^59^ and implemented the Paetkau *et al*.^60^ simulation algorithm, with the number of individuals set to 100,000 and the alpha value set to 0.005.

## Results

**Figure 2:**
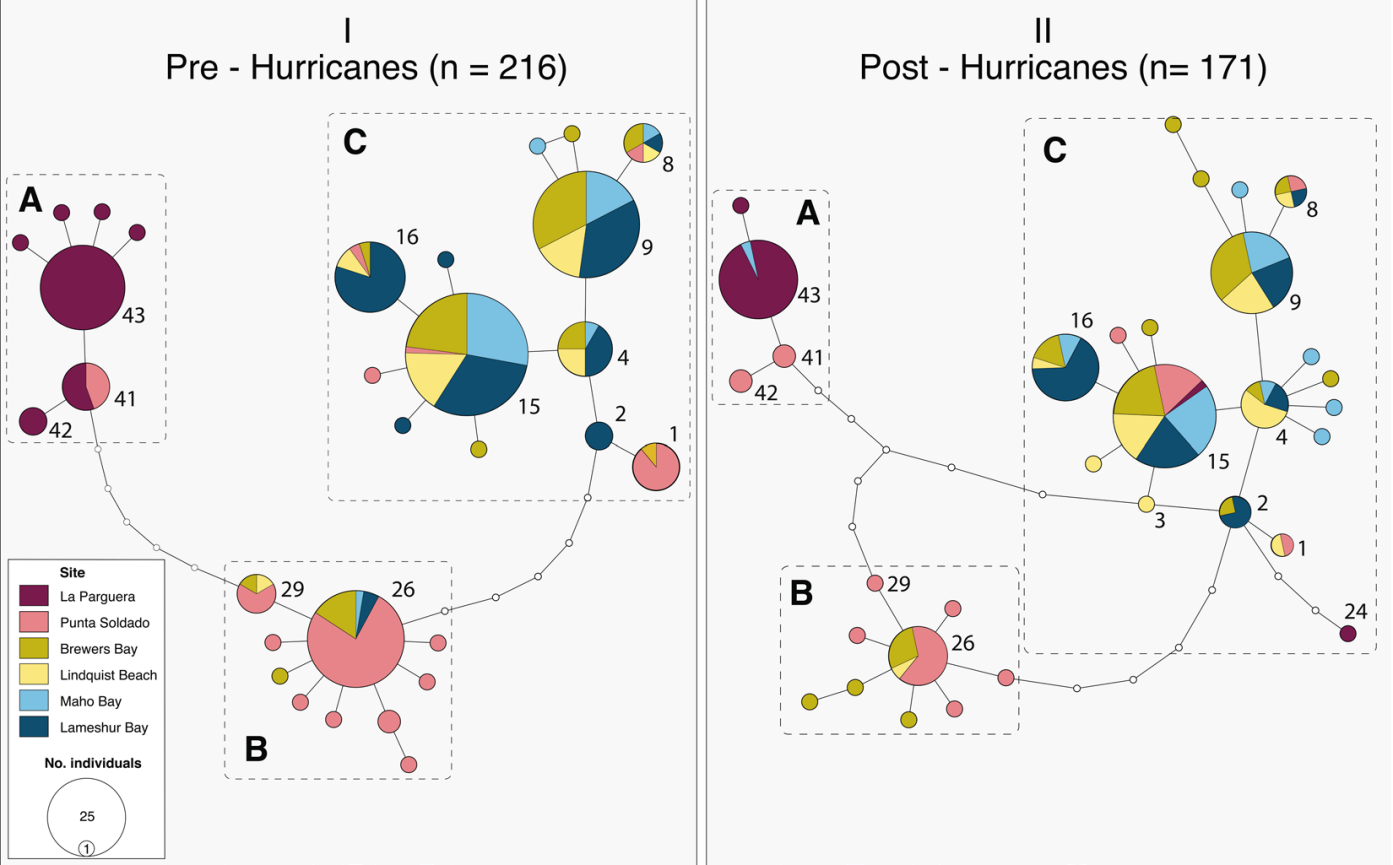
Haplotype networks representing the genetic diversity of *Gnathia marleyi* and its spatial distribution before and after the 2017 hurricanes. Pre- and post-hurricane datasets are indicated by panels I and II, respectively. The haplotypes found in both pre- and post-hurricane datasets are indicated by haplotype number. Haplogroups A, B, and C are indicated by dashed boxes.

In total, 432 gnathiids were sequenced across sites and years (Table 1). Overall, genetic diversity was highest at the center of the study area and lower toward the margins. Punta Soldado and Brewers Bay generally showed higher genetic diversity such as absolute number of haplotypes, Pi, and nucleotide diversity, while La Parguera and Lameshur Bay showed the lowest overall diversity (Table 1). On the other hand, evenness was highest in Lameshur Bay but decreased along awesterly gradient. Pre-hurricane haplotype networks revealed three main haplogroups (A–C; Fig. 2), differing by 5 to 12 mutations. Haplogroup A contained seven haplotypes, which were exclusive to La Parguera with the exception of one haplotype (hap_41) that was shared with Punta Soldado. Haplogroup B had ten haplotypes and was predominantly represented by individuals from Punta Soldado. Haplogroup C was comprised of 13 haplotypes shared between sample collections from the U.S. Virgin Islands, with one haplotype found predominantly in Punta Soldado. The two most common gnathiid haplotypes (hap_09 and hap_15) belonged to haplogroup C.

**Table 1:** Summary of molecular diversity indices per site and per year. Geographic regions are indicated above each site.

Molecular diversity indices	W. Puerto Rico			E. Puerto Rico			U.S. Virgin Islands								
	La Parguera			Punta Soldado			Brewers Bay		Lindquist Beach		Maho Bay		Lameshur Bay		
Year	2014	2016	2018	2013	2016	2018	2016	2018	2017	2018	2017	2018	2016	2017	2018
n	24	17	27	32	25	28	46	35	24	24	29	24	34	30	33
No. of haplotypes	5	3	4	11	9	12	11	14	6	9	6	9	7	8	6
Expected no. of haplotypes	4.12	3.00	2.89	7.28	7.19	8.57	6.53	8.67	5.32	7.25	4.34	7.17	5.51	6.19	5.18
Evenness	0.60	0.63	0.43	0.52	0.55	0.65	0.68	0.67	0.76	0.76	0.64	0.65	0.78	0.78	0.82
Pi	0.59	0.74	1.44	4.11	3.47	5.37	3.35	3.96	1.81	2.00	1.59	2.11	1.81	1.97	1.53
Haplotype diversity	0.53	0.40	0.21	0.73	0.72	0.84	0.79	0.86	0.75	0.83	0.60	0.78	0.77	0.81	0.77
Nucleotide diversity	0.0011	0.0014	0.0028	0.0079	0.0067	0.0104	0.0065	0.0077	0.0035	0.0039	0.0031	0.0041	0.0035	0.0038	0.003

The post-hurricane haplotype network revealed the same three haplogroups but the overall topology of the network changed, with haplogroups now differing only by 4 to 5 mutations. The reticulation in the post-hurricane haplotype network was the result of a new haplotype (hap_03) recovered in Lindquist Beach, which connected haplogroups A and C by five mutation steps. The most notable changes in haplotype distribution were the presence of haplogroup A sequences (hap_43) in Maho Bay (previously found only in Puerto Rico), and of haplogroup C sequences (hap_15 and hap_24) in La Parguera (previously absent from this location). Haplogroup B was still predominantly represented by individuals from Punta Soldado, however an increase in individuals collected at this locality and assigned to haplogroups A and C was observed. After the 2017 hurricane season, the overall proportion of haplotypes belonging to group C increased substantially (pre-hurricane: 43.3%, post-hurricane: 59.4%), mostly driven by an increase in singleton haplotypes. Moreover, after the 2017 hurricanes, a greater proportion of individuals collected from the U.S. Virgin Islands had haplotypes that clustered into groups A and B, and the most abundant haplotype overall (hap_15) had representatives from every site sampled (Supplementary information, Table S1).

PERMANOVAs showed that prior to the 2017 hurricanes, the genetic diversity indices did not differ between years within sites (see Table S2 in Supporting Information). PERMANOVA results also showed that the molecular diversity indices were significantly different between sites and that effect of the hurricanes was significant and increased the expected number of haplotypes, Pi, and nucleotide diversity within sites (see Table S3). In addition, the interaction between Disturbance and Site had a significant effect in haplotype evenness (R^2^ = 0.21, p = 0.01), indicating that the impact of hurricanes on evenness varied among sites (see Table S3).

Within-site pairwise PhiST estimates did not differ between years under undisturbed conditions (p > 0.05; see Table S4) or between pre- and post-hurricane datasets (p > 0.05; see Table S4). On the other hand, among-site pairwise PhiST estimates pre-hurricanes showed significant genetic divergence between La Parguera and all other sample collections (Table 2). Similarly, PhiSTs estimated for Punta Soldado were also significantly different from all other sample collections although values were lower than those estimated for La Parguera. Within the U.S. Virgin Islands low but significant differences were found only between Brewers Bay and Lameshur Bay (p < 0.05). The same overall pattern of among-site genetic differentiation was observed after the 2017 hurricanes, with individuals from La Parguera and Punta Soldado significantly differentiated from all other sites. However, nearly all pairwise PhiST estimates decreased after the hurricanes with the only increase in differentiation found between Brewers Bay and Lameshur Bay (see Fig. S1).

**Table 2:** Summary of pairwise PhiST values among all sampling localities. Pairwise comparisons that are statistically significant are indicated with an asterisk.

Pre- and post-hurricane pairwise PhiST						
Pre-hurricanes						
	Parguera	Soldado	Brewers	Lindquist	Maho	Lameshur
Parguera	—					
Soldado	0.691*	—				
Brewers	0.768*	0.428*	—			
Lindquist	0.876*	0.555*	0.027	—		
Maho	0.880*	0.575*	0.042	0.000	—	
Lameshur	0.843*	0.597*	0.076*	0.000	0.015	—
**Post-hurricanes**						
Parguera	—					
Soldado	0.534*	—				
Brewers	0.680*	0.187*	—			
Lindquist	0.800*	0.339*	0.028	—		
Maho	0.788*	0.358*	0.058	0.000	—	
Lameshur	0.824*	0.426*	0.119*	0.061	0.029	—

For both pre- and post-hurricane datasets, the scenario a) of global panmixia among *G. marleyi* sample collections was rejected by AMOVA results (FST pre:0.581 and post:0.474;  p < 0.05, Table 3), indicating significant spatial genetic structure in the data. Scenario b) of population structure according to regional groups showed significant among-group differences before the 2017 hurricanes (FCT pre:0.688; p < 0.05), but not after the hurricanes (FCT post:0.578, p > 0.05; Table 3). Additionally, differences among-sites within group were not significant pre-hurricanes (FSC pre:0.017; p > 0.05) but were significant in post-hurricane datasets (post:0.050,  p < 0.05; Table 3). This latter result is likely driven by the increased differentiation between Lameshur Bay and Brewers Bay (Table 2). Overall, the percent of genetic variation explained by among group differences decreased from 68.8% to 57.8% after the hurricanes, and the percent of variation explained by within-sites variability increased from 30.7% to 40.1% (Table 3).

**Table 3:** Summary of AMOVA results. AMOVAs testing panmixia (a) included a single group consisting of all sampling sites. AMOVAs testing structure at the regional level (b) are grouped by group1: La Parguera, group2: Punta Soldado, and group3: Brewers Bay, Lindquist Beach, Maho Bay, and Lameshur Bay. Asterisk indicate significant genetic structure at a given level.

Pre- and post-hurricane AMOVAs		Pre Hurricanes				Post Hurricanes			
Scenario	Source of Variation	Percentage variation	Fixation index	value	p - value	Percentage variation	Fixation index	value	p - value
a) Panmixia	Among groups	58.05	FCT	—	—	47.36	FCT	—	—
	Among populations within groups	—	FSC	—	—	—	FSC	—	—
	Within populations	41.95	FST	0.581	<0.001*	52.64	FST	0.474	<0.001*
b) Regions	Among groups	68.77	FCT	0.688	0.003*	57.76	FCT	0.578	0.064
	Among populations within groups	0.53	FSC	0.017	0.075	2.11	FSC	0.050	0.002*
	Within populations	30.71	FST	0.693	<0.001*	40.12	FST	0.599	<0.001*

**Figure 3:**
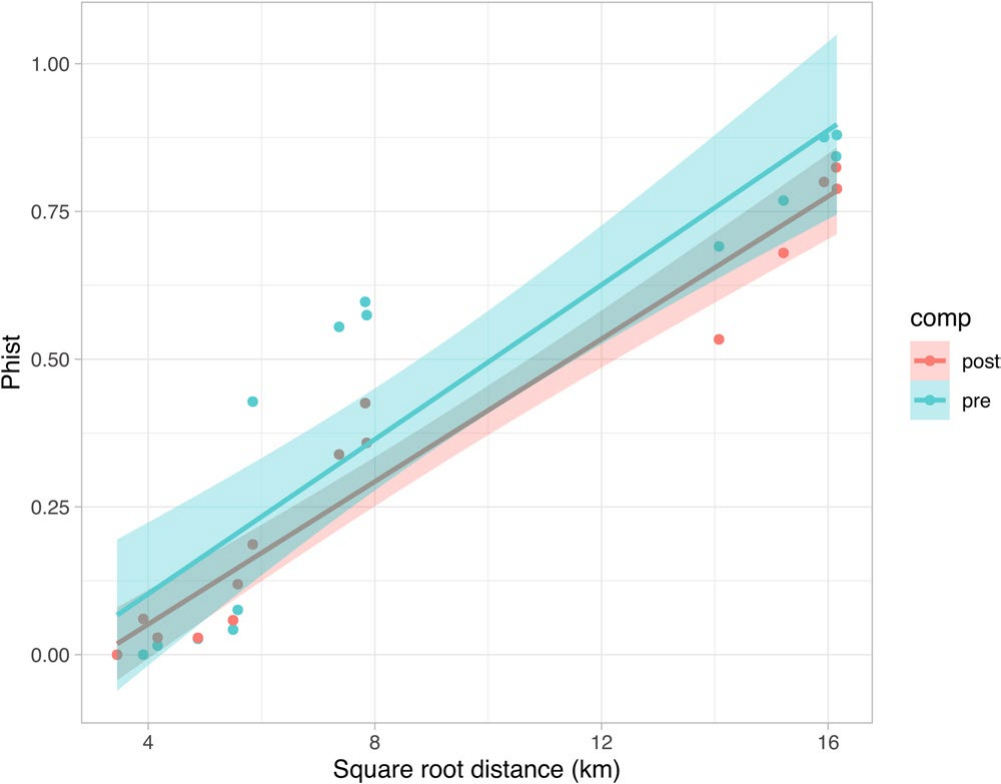
Scatter plot illustrating the relationship between geographic distance and genetic distance (Pairwise PhiST). Shaded region indicates the 95% confidence interval for the adjusted R^2^.

The linear regression between genetic and geographic distances was significant for both pre- and post- hurricane datasets (p < 0.05, adjusted R^2^ = 0.82, 0.94, respectively), suggesting a clear and consistent pattern of IBD in *G. marleyi* (Fig. 3). The Geneclass2 analysis detected seven first-generation migrants in the entire dataset with probabilities < 0.005. Additionally it showed somewhat distinct patterns of gene flow before and after the hurricanes (Supplemental Table S5). Before the 2017 hurricane season, two first-generation migrants were detected between Punta Soldado and Brewers Bay and one between Lameshur Bay and Punta Soldado, with gene flow occurring in opposite directions along 34–57 km (Table S5). In turn, four first-generation migrants were detected after the hurricanes between Punta Soldado and Lindquist Beach (n = 1), Lameshur Bay and Brewers Bay (n = 1), and between the two most distant sampling localities, La Parguera and Maho Bay (n = 2). Gene flow after the 2017 hurricanes occurred also in both eastward as well as westward directions and over distances of 31–260 km.

## Discussion

In accordance with our expectations, the results from the present work confirm that *G. marleyi* exhibits generally limited dispersal ability, with genetic differentiation between sample collections conforming to a pattern of isolation by distance. Specifically, prior to the 2017 hurricane season, gnathiid sample collections were genetically differentiated at the regional level (>100 km), with geographically distant locations showing the largest genetic distances (i.e. La Parguera vs. sites in the U.S. Virgin Islands; Fig. 3). At the local level (<30 km, i.e. within the US Virgin Islands), no significant differences were detected among sites except between Lameshur and Brewers Bay, the two most distant sites within that region. Based on our data, under stable environmental conditions, gene-flow among gnathiid populations appears to be limited to around 40 km. Indeed, the first-generation migrants detected prior to the hurricanes only dispersed among Punta Soldado and the U.S. Virgin Islands sites, and samples were generally included in separate, well resolved haplogroups. In contrast, following the 2017 hurricane season, individuals were displaced between La Parguera and Maho Bay, a distance of more than 250 km. These results strongly suggest that hurricanes mediate long distance gene flow in this species, but contradict our original hypothesis of westward migration following the hurricane trajectories. Although the three main haplogroups were maintained following the 2017 storms, there was enough gene flow to substantially increase the genetic heterogeneity within sites and have a homogenizing effect among regional groups (i.e. increased diversity shared among sites and among groups).

Previous studies on the impacts of other types of environmental disturbance on other coastal species have also found that genetic diversity can be maintained or even increased after major disturbances. For instance, genetic diversity of marine iguanas was maintained despite high mortalities following the El Niño Southern Oscillation in 1998^61^. Similarly, genetic diversity of intertidal gobies did not change after the record earthquake and subsequent tsunami that struck eastern Japan in 2011^26^. Interestingly, although within-population abundances of a widespread intertidal mud snail, *Batillaria attramentaria*, showed strong declines after the 2011 tsunami, the direct impact of this disturbance on genetic diversity is difficult to assess given the large temporal variability observed in both pre- and post-tsunami conditions^27^. Only one previous study attempted to examine the effects of a tropical cyclone on the genetic structure of an aquatic organism^28^. In that study, the authors compared genetic diversity of a single population of the estuarine poeciliid fish *Poecilia latipinna* obtained with microsatellite data after hurricane Dennis in 2005, with those obtained with alloenzyme data 20 years prior^62^. In their study, they observed an increase in genetic diversity and reduced population differentiation which they attributed to the storm surge driven by the cyclone. However, the 20-year time lag between two sampling points and the differences in molecular markers made it difficult to link this change to the hurricane itself.

While the above-mentioned studies provided important insights regarding the potential impacts of disturbances on gene flow of marine taxa, they focused on species with innate potential for long distance dispersal, and thus theoretically more resilient to disturbance. Importantly, only Miura *et al*.^27^ and the present study included pre-disturbance temporal replication and estimates of differentiation for multiple populations. In the present work, annual sample collections allowed the analysis of non-overlapping generations of *G. marleyi*, consequently, we were able to unequivocally assess the effects that major hurricanes can have on the genetic diversity patterns of regional endemics with limited dispersal.

Although our study focused on a single species, inferences gained herein may extend to other species with similar ecologies and geographic distribution. For instance, otherwise dispersal-limited marine coastal species may benefit from increased and more frequent long distance dispersal events in regions periodically affected by tropical cyclones, especially since storm intensity and frequency is expected to increase due to ongoing climate change^63,64^. Ultimately, such events can contribute to changes in geographic distribution and the evolutionary potential of these species. While increased gene-flow and genetic diversity may buffer species from the loss of alleles due to genetic drift following stochastic demographic events, such as population bottlenecks^65^, these same processes may also dilute or even displace locally adapted alleles^66,67^. Aside from potentially impacting evolutionary outcomes of species, major disturbances may more immediately influence ecological interactions. In the case of parasitic gnathiid isopods, in addition to the direct effects on hosts via tissue damage and blood loss^33^, they can also function as vectors of diseases^68–70^. Understanding the spatial scales at which these potential vectors may spread and how major disturbances might alter such patterns is therefore critical.

The work presented here characterizes the acute effects of a severe climatic disturbance on the genetic diversity and structure of a coastal marine species. We demonstrate that major hurricanes can increase genetic diversity and reduce genetic differentiation locally and regionally, in a low-dispersal coral reef species. Besides tropical cyclones, coral reef ecosystems face numerous other threats prompted by global climate change, such as bleaching events, rising sea levels, and increased ocean acidification^71–73^ and these are also known to impact the ecology of gnathiids^74^. Clearly, more molecular studies are needed to address how these disturbances can affect other members of the coral reef community, including cryptic species, to better understand their potential resilience to future disturbances.

## Supplementary information

Supplementary information 

## Data Availability

The genetic data used in this manuscript are available at Genbank under accession numbers MT186550-MT186597.
