# Supplementary material for: Hurricane-induced disturbance increases genetic diversity and population admixture of the direct-brooding isopod, Gnathia marleyi
Source: Sci Rep. 2020 May 26;10:8649. doi: 10.1038/s41598-020-64779-7 (PMC7250855; doi:10.1038/s41598-020-64779-7)
Supplement: Supplementary file 1 — Supplementary information [file 41598_2020_64779_MOESM1_ESM.docx]

Hurricane-induced disturbance increases genetic diversity and population admixture of the direct-brooding isopod, *Gnathia marleyi*.

J. Andrés Pagán^1^, Ana Veríssimo^1^, Paul C. Sikkel^2,3*^, Raquel Xavier^1*^

1. CIBIO- Universidade do Porto, Centro de Investigção em Biodiversidade e Recursos Genéticos, Campus Agrário de Vairão, Rua Padre Armando Quintas, 4485-661 Vairão, Portugal

2. Department of Biological Sciences and Environmental Sciences Program, Arkansas State University, PO Box 599, State University, AR 72467

3. Water Research Group, Unit for Environmental Sciences and Management, North-West University, Potchefstroom, South Africa

e-mail addresses: j.andres.pagan@gmail.com; averissimo@cibio.up.pt; psikkel@astate.edu raq.xavier@cibio.up.pt

*corresponding authors: Paul C. Sikkel and Raquel Xavier

Table S1. Summary of haplotypes found at each site before and after the 2017 North Atlantic Hurricanes.

| Haplotype frequencies | | |  |  |  |  |  |  |  |  |  |  |  |
| --- | --- | --- | --- | --- | --- | --- | --- | --- | --- | --- | --- | --- | --- |
| Haplotype | Parguera | | Soldado | | Brewers | | Lindquist | | Maho | | Lameshur | | Accession Number |
|  | Pre | Post | Pre | Post | Pre | Post | Pre | Post | Pre | Post | Pre | Post |  |
| 1 | - | - | 8 | 1 | 1 | - | - | 1 | - | - | - | - | MT186550 |
| 2 | - | - | - | - | - | 1 | - | - | - | - | 3 | 3 | MT186551 |
| 3 | - | - | - | - | - | - | - | 1 | - | - | - | - | MT186561 |
| 4 | - | - | - | - | 3 | 1 | 3 | 5 | 1 | 1 | 5 | 2 | MT186552 |
| 5 | - | - | - | - | - | - | - | - | - | 1 | - | - | MT186553 |
| 6 | - | - | - | - | - | - | - | - | - | 1 | - | - | MT186570 |
| 7 | - | - | - | - | - | 1 | - | - | - | - | - | - | MT186554 |
| 8 | - | - | 1 | 1 | 2 | 1 | 1 | 1 | 1 | - | 1 | 1 | MT186555 |
| 9 | - | - | - | - | 15 | 9 | 7 | 6 | 8 | 6 | 16 | 6 | MT186556 |
| 10 | - | - | - | - | - | - | - | - | 1 | - | - | - | MT186559 |
| 11 | - | - | - | - | - | - | - | - | - | 1 | - | - | MT186560 |
| 12 | - | - | - | - | - | 1 | - | - | - | - | - | - | MT186557 |
| 13 | - | - | - | - | - | 1 | - | - | - | - | - | - | MT186558 |
| 14 | - | - | 1 | 1 | - | - | - | - | - | - | - | - | MT186563 |
| 15 | - | 1 | 1 | 7 | 14 | 9 | 10 | 7 | 17 | 10 | 19 | 9 | MT186562 |
| 16 | - | - | 1 | - | 1 | 3 | 2 | 1 | - | 2 | 16 | 12 | MT186564 |
| 17 | - | - | - | - | - | - | - | - | - | - | 1 | - | MT186565 |
| 18 | - | - | - | - | - | - | - | - | - | - | 1 | - | MT186566 |
| 19 | - | - | - | - | - | - | - | 1 | - | - | - | - | MT186567 |
| 20 | - | - | - | - | 1 | - | - | - | - | - | - | - | MT186568 |
| 21 | - | - | - | - | - | 1 | - | - | - | - | - | - | MT186569 |
| 22 | - | - | - | - | 1 | - | - | - | - | - | - | - | MT186571 |
| 23 | - | - | - | - | - | - | - | - | - | 1 | - | - | MT186572 |
| 24 | - | 1 | - | - | - | - | - | - | - | - | - | - | MT186573 |
| 25 | - | - | 1 | - | - | - | - | - | - | - | - | - | MT186574 |
| 26 | - | - | 29 | 9 | 6 | 4 | - | 1 | 1 | - | 2 | - | MT186575 |
| 27 | - | - | 1 | - | - | - | - | - | - | - | - | - | MT186586 |
| 28 | - | - | 1 | 1 | - | - | - | - | - | - | - | - | MT186576 |
| 29 | - | - | 4 | 1 | 1 | - | 1 | - | - | - | - | - | MT186577 |
| 30 | - | - | 1 | - | - | - | - | - | - | - | - | - | MT186578 |
| 31 | - | - | - | 1 | - | - | - | - | - | - | - | - | MT186579 |
| 32 | - | - | - | 1 | - | - | - | - | - | - | - | - | MT186580 |
| 33 | - | - | - | 1 | - | - | - | - | - | - | - | - | MT186587 |
| 34 | - | - | - | - | 1 | - | - | - | - | - | - | - | MT186588 |
| 35 | - | - | - | - | - | 1 | - | - | - | - | - | - | MT186581 |
| 36 | - | - | 2 | - | - | - | - | - | - | - | - | - | MT186584 |
| 37 | - | - | 1 | - | - | - | - | - | - | - | - | - | MT186585 |
| 38 | - | - | - | - | - | 1 | - | - | - | - | - | - | MT186582 |
| 39 | - | - | - | - | - | 1 | - | - | - | - | - | - | MT186583 |
| 40 | - | - | 1 | - | - | - | - | - | - | - | - | - | MT186589 |
| 41 | 5 | - | 4 | 2 | - | - | - | - | - | - | - | - | MT186590 |
| 42 | 3 | - | - | 2 | - | - | - | - | - | - | - | - | MT186591 |
| 43 | 29 | 24 | - | - | - | - | - | - | - | 1 | - | - | MT186594 |
| 44 | 1 | - | - | - | - | - | - | - | - | - | - | - | MT186593 |
| 45 | 1 | - | - | - | - | - | - | - | - | - | - | - | MT186592 |
| 46 | 1 | - | - | - | - | - | - | - | - | - | - | - | MT186596 |
| 47 | 1 | - | - | - | - | - | - | - | - | - | - | - | MT186597 |
| 48 | - | 1 | - | - | - | - | - | - | - | - | - | - | MT186595 |

Table S2. Summary of PERMANOVAs testing the effects of Year on molecular diversity indices for localities with pre-hurricane temporal replication.

PERMANOVA: Year

| Molecular diversity index | Factor | Df | Sum of Sq. | R2 | F | Pr(>F) |
| --- | --- | --- | --- | --- | --- | --- |
| Number of haplotypes | Year | 1 | 3.54 | 0.09 | 0.38 | 0.60 |
|  | Residual | 4 | 37.29 | 0.91 |  |  |
|  | Total | 5 | 40.83 | 1.00 |  |  |
| Expected number of haplotypes | Year | 1 | 0.25 | 0.02 | 0.07 | 0.80 |
|  | Residual | 4 | 14.39 | 0.98 |  |  |
|  | Total | 5 | 14.64 | 1.00 |  |  |
| Evenness | Year | 1 | 0.03 | 0.47 | 3.57 | 0.11 |
|  | Residual | 4 | 0.03 | 0.53 |  |  |
|  | Total | 5 | 0.06 | 1.00 |  |  |
| Pi | Year | 1 | 0.84 | 0.08 | 0.36 | 0.63 |
|  | Residual | 4 | 9.29 | 0.92 |  |  |
|  | Total | 5 | 10.13 | 1.00 |  |  |
| Haplotype diversity | Year | 1 | 0.00 | 0.03 | 0.11 | 0.80 |
|  | Residual | 4 | 0.12 | 0.97 |  |  |
|  | Total | 5 | 0.13 | 1.00 |  |  |
| Nucleotide diversity | Year | 1 | 0.00 | 0.08 | 0.36 | 0.63 |
|  | Residual | 4 | 0.00 | 0.92 |  |  |
|  | Total | 5 | 0.00 | 1.00 |  |  |

Table S3. Summary of PERMANOVAs testing the effect of site and disturbance (Hurricanes) on the different molecular diversity indices. Asterisks indicate factors that significantly impact molecular diversity index.

PERMANOVA: Site and Disturbance

| Molecular Diversity Index | Factor | Df | Sum Of Sqs | R2 | F | Pr(>F) |
| --- | --- | --- | --- | --- | --- | --- |
| Number of Haplotypes | Disturbance | 1 | 10.00 | 0.07 | 6.67 | 0.08 |
|  | Site | 5 | 110.51 | 0.81 | 14.73 | 0.02* |
|  | Disturbance:Site | 5 | 10.99 | 0.08 | 1.47 | 0.39 |
|  | Residual | 3 | 4.50 | 0.03 |  |  |
|  | Total | 14 | 136.00 | 1.00 |  |  |
| Expected number of haplotypes | Disturbance | 1 | 4.55 | 0.10 | 15.82 | 0.02* |
|  | Site | 5 | 34.37 | 0.74 | 23.91 | 0.01* |
|  | Disturbance:Site | 5 | 6.55 | 0.14 | 4.56 | 0.10 |
|  | Residual | 3 | 0.86 | 0.02 |  |  |
|  | Total | 14 | 46.33 | 1.00 |  |  |
| Evenness | Disturbance | 1 | 0.00 | 0.00 | 0.13 | 0.74 |
|  | Site | 5 | 0.13 | 0.79 | 112.93 | 0.00* |
|  | Disturbance:Site | 5 | 0.03 | 0.21 | 29.51 | 0.01* |
|  | Residual | 3 | 0.00 | 0.00 |  |  |
|  | Total | 14 | 0.17 | 1.00 |  |  |
| Pi | Disturbance | 1 | 1.19 | 0.05 | 15.63 | 0.03* |
|  | Site | 5 | 22.90 | 0.89 | 60.16 | 0.01* |
|  | Disturbance:Site | 5 | 1.36 | 0.05 | 3.57 | 0.16 |
|  | Residual | 3 | 0.23 | 0.01 |  |  |
|  | Total | 14 | 25.67 | 1.00 |  |  |
| Haplotype diversity | Disturbance | 1 | 0.01 | 0.01 | 1.97 | 0.30 |
|  | Site | 5 | 0.38 | 0.81 | 27.13 | 0.04* |
|  | Disturbance:Site | 5 | 0.07 | 0.16 | 5.23 | 0.20 |
|  | Residual | 3 | 0.01 | 0.02 |  |  |
|  | Total | 14 | 0.47 | 1.00 |  |  |
| Nucleotide diversity | Disturbance | 1 | 0.00 | 0.05 | 15.58 | 0.04* |
|  | Site | 5 | 0.00 | 0.89 | 60.01 | 0.01* |
|  | Disturbance:Site | 5 | 0.00 | 0.05 | 3.56 | 0.16 |
|  | Residual | 3 | 0.00 | 0.01 |  |  |
|  | Total | 14 | 0.00 | 1.00 |  |  |

Table S4. Summary of pairwise PhiSTs for sites with pre-hurricane temporal replication. Boxed comparisons highlight within site comparisons.

PhiST and temporal variation

| Pairwise PhiST | |  |  |  |  |  |
| --- | --- | --- | --- | --- | --- | --- |
|  | Parguera14 | Parguera16 | Soldado13 | Soldado16 | Lameshur16 | Lameshur17 |
| Parguera14 | - |  |  |  |  |  |
| Parguera16 | 0.012 | - |  |  |  |  |
| Soldado13 | 0.676 | 0.651 | - |  |  |  |
| Soldado16 | 0.729 | 0.703 | 0.000 | - |  |  |
| Lameshur16 | 0.850 | 0.838 | 0.564 | 0.648 | - |  |
| Lameshur17 | 0.848 | 0.834 | 0.544 | 0.631 | 0.000 | - |
|  |  |  |  |  |  |  |
| P-values |  |  |  |  |  |  |
|  | Parguera14 | Parguera16 | Soldado13 | Soldado16 | Lameshur16 | Lameshur17 |
| Parguera14 | - |  |  |  |  |  |
| Parguera16 | 0.894 | - |  |  |  |  |
| Soldado13 | 0.000 | 0.000 | - |  |  |  |
| Soldado16 | 0.000 | 0.000 | 0.960 | - |  |  |
| Lameshur16 | 0.000 | 0.000 | 0.000 | 0.000 | - |  |
| Lameshur17 | 0.000 | 0.000 | 0.000 | 0.000 | 0.960 | - |

Table S5. Summary of the seven first-generation migrants identified. Source populations are based on sampling site. Values are of the log home/ log max likelihood with the lowest value indicating the most likely population of origin.

First-generation migrants

| Assigned sample | Home | Source | Distance (km) | Direction | Probability | Parguera | Soldado | Brewers | Lindquist | Maho | Lameshur |
| --- | --- | --- | --- | --- | --- | --- | --- | --- | --- | --- | --- |
| *Pre-Hurricanes* |  |  |  |  |  |  |  |  |  |  |  |
| PRe1325 | Soldado | Lameshur | 61 | east - west | 0 | 3.5201 | 3.6107 | 1.3095 | 1.2101 | 1.4269 | 0.5437 |
| SthW1641 | Brewers | Soldado | 34 | west - east | 0.00031 | 3.5201 | 0.9793 | 3.5897 | 1.6812 | 3.4136 | 3.6725 |
| SthW1655 | Brewers | Soldado | 34 | west - east | 0.00456 | 3.5201 | 1.2337 | 3.5897 | 1.6812 | 3.4136 | 3.6725 |
| *Post-Hurricanes* |  |  |  |  |  |  |  |  |  |  |  |
| PRw1806 | Parguera | Maho | 260 | east - west | 0 | 3.5138 | 1.0303 | 0.5517 | 0.4592 | 0.3007 | 0.5437 |
| SjnN1829 | Maho | Parguera | 260 | west - east | 0 | 0.1144 | 3.6157 | 3.5951 | 3.3714 | 3.4055 | 3.6725 |
| SthN1824 | Lindquist | Soldado | 54 | west - east | 0 | 3.5201 | 0.3545 | 0.9129 | 3.3625 | 1.7234 | 1.6857 |
| SthW1817 | Brewers | Lameshur | 31 | east - west | 0.00389 | 3.5201 | 3.6157 | 3.5897 | 3.3714 | 3.4136 | 1.2116 |

Figure S1. Scatter plot illustrating the pairwise PhiST values pre- and post-hurricanes observed at a given pair of sampling collections with labeled points indicating significantly different comparisons. Points that fall below the diagonal had decreased PhiST following the 2017 hurricanes.
